# Testosterone Supplementation Induces Age-Dependent Augmentation of the Hypoxic Ventilatory Response in Male Rats With Contributions From the Carotid Bodies

**DOI:** 10.3389/fphys.2021.781662

**Published:** 2021-12-24

**Authors:** Tara A. Janes, Danuzia Ambrozio-Marques, Sébastien Fournier, Vincent Joseph, Jorge Soliz, Richard Kinkead

**Affiliations:** ^1^Department of Physiology, Women and Children’s Health Research Institute, University of Alberta, Edmonton, AB, Canada; ^2^Department of Pediatrics, Québec Heart and Lung Institute, Université Laval, Québec, QC, Canada; ^3^Department of Surgery, Faculty of Medicine, Université Laval, Québec, QC, Canada

**Keywords:** carotid body, hypoxia, testosterone, sleep apnea, respiration, nucleus of the solitary tract

## Abstract

Excessive carotid body responsiveness to O_2_ and/or CO_2_/H^+^ stimuli contributes to respiratory instability and apneas during sleep. In hypogonadal men, testosterone supplementation may increase the risk of sleep-disordered breathing; however, the site of action is unknown. The present study tested the hypothesis that testosterone supplementation potentiates carotid body responsiveness to hypoxia in adult male rats. Because testosterone levels decline with age, we also determined whether these effects were age-dependent. *In situ* hybridization determined that androgen receptor mRNA was present in the carotid bodies and caudal nucleus of the solitary tract of adult (69 days old) and aging (193–206 days old) male rats. In urethane-anesthetized rats injected with testosterone propionate (2 mg/kg; i.p.), peak breathing frequency measured during hypoxia (FiO_2_ = 0.12) was 11% greater vs. the vehicle treatment group. Interestingly, response intensity following testosterone treatment was positively correlated with animal age. Exposing *ex vivo* carotid body preparations from young and aging rats to testosterone (5 nM, free testosterone) 90–120 min prior to testing showed that the carotid sinus nerve firing rate during hypoxia (5% CO_2_ + 95% N_2_; 15 min) was augmented in both age groups as compared to vehicle (<0.001% DMSO). Ventilatory measurements performed using whole body plethysmography revealed that testosterone supplementation (2 mg/kg; i.p.) 2 h prior reduced apnea frequency during sleep. We conclude that in healthy rats, age-dependent potentiation of the carotid body’s response to hypoxia by acute testosterone supplementation does not favor the occurrence of apneas but rather appears to stabilize breathing during sleep.

## Introduction

The carotid bodies (CB’s) glomus cells (type I) have long been known as the body’s main O_2_ sensors (Ortega-Sáenz and López-Barneo, 2020). In light of recent evidence, however, they are now appreciated for their ability to “sense and respond” to a variety of non-respiratory stimuli including angiotensin II, leptin, cytokines, insulin, and lactate (Conde et al., 2014, 2020; Torres-Torrelo et al., 2021). Their strategic location at the bifurcation of the common carotid arteries allows them to act as multimodal chemosensors and thus play multiple roles in homeostasis beyond respiratory control. That being said, CB dysfunction remains of utmost importance in the pathophysiology of sleep-disordered breathing (SDB) (Smith et al., 2003; Prabhakar, 2016; Ortega-Sáenz and López-Barneo, 2020; Iturriaga et al., 2021), a complex and multifactorial respiratory disorder. Augmented carotid body responsiveness to fluctuations in arterial blood gases during sleep leads to excessive ventilatory adjustments, and promotes the recurrence of apneas. Abnormal carotid body function also contributes to hypertension, an important comorbidity of SDB (Paton et al., 2013; Del Rio et al., 2016; Narkiewicz et al., 2016; Iturriaga et al., 2021). Animal models of SDB reproducing the recurrent drops in arterial O_2_ show that chronic intermittent hypoxia augments the CB’s chemosensitivity and reproduce key cardiorespiratory comorbidities observed in SDB patients (Dempsey et al., 2010; Puri et al., 2021). However, chronic intermittent hypoxia is a consequence of SDB and the periodic total or partial obstruction of the upper airway is not reached in this model. Thus, the origins of SDB (including CB dysfunction) remain elusive.

Sleep disordered breathing is consistently reported with higher prevalence in men than pre-menopausal women (Young et al., 1993; Coutinho Costa et al., 2019). The growing appreciation for the sexual dimorphism of the manifestations of this disease has re-ignited the interest in the influence of sex hormones in cardiorespiratory control (Behan and Kinkead, 2011; Kinkead et al., 2021; Tenorio-Lopes and Kinkead, 2021). Data from animal models suggest that testosterone acting (at least in part) at the level of the CB facilitates ventilatory responses to hypoxia. In neutered cats, testosterone treatment enhances the hypoxic ventilatory response (HVR) and augments carotid sinus nerve (CSN) output during hypoxemia (Tatsumi et al., 1994). In adult male rats, surgical reduction of testosterone by gonadectomy blunts the ventilatory response to hypoxia, but only in animals subjected to neonatal stress (Fournier et al., 2014). Human data are, however, more enigmatic. Testosterone supplementation in hypogonadal men was reported to increase or decrease the HVR [(Matsumoto et al., 1985) vs. (White et al., 1985), respectively]. Despite those conflicting results, testosterone replacement in hypogonadal men increased ventilation during restful breathing and worsens sleep-related apneas/hypopneas (Matsumoto et al., 1985; White et al., 1985; Liu et al., 2003; Andersen and Tufik, 2008; Hoyos et al., 2012). As a result, testosterone replacement therapy is currently contraindicated for men exhibiting SDB. Yet, many studies have found that testosterone levels in SDB patients are lower than those of healthy subjects (Luboshitzky et al., 2002; Mohammadi et al., 2019). These puzzling observations highlight the fact that our knowledge of the basic actions of testosterone on respiratory control are limited which makes it difficult to determine if this hormone plays a role in the pathophysiology of SDB.

To further our basic understanding of the role of testosterone in respiratory control, we tested the hypothesis that acute testosterone injection enhances the HVR by potentiating CB responsiveness to hypoxia. Given that testosterone levels decline with age, we performed experiments on young and aging male rats to test for age-dependent effects. To do so, we first established the presence of androgen receptor mRNA in the CB using RNAscope^®^. We then used an anesthetized rat preparation to measure the effects of acute (hours) testosterone injection on the HVR; this approach facilitates standardization of the hypoxic stimulus with strict monitoring of arterial blood gases. The action of testosterone on CB responsiveness to hypoxia was measured electrophysiologically using *ex vivo* preparations. Lastly, we evaluated the impacts of testosterone supplementation under physiologically relevant conditions by measuring the occurrence of apneas in naturally sleeping animals.

## Materials and Methods

### Animals and Ethical Approval

Physiological experiments (Series II, III, and IV) were performed at the Québec Heart and Lung Institute (Université Laval, Québec, QC, Canada) on adult male Sprague-Dawley rats. The animals used were born to primiparous females that were obtained from Charles River and bred in house. Following birth, rats were raised in our animal care facilities until they were used for experimentation. Anatomical experiments (Series I) were performed at the Women and Children’s Health Research Institute (University of Alberta, Edmonton, AB, Canada) on adult male rats born and raised in the University of Alberta Health Sciences Animal Care Facility. Specifically, pregnant dams were purchased (Charles River) and gave birth in the animal facility 10–14 days after arrival. In all experiments, rats were supplied with food and water *ad libitum* and maintained under standard housing conditions of 21°C, with a 12:12 dark:light cycle. The number of animals, age range, and body weights of the rats are detailed in each series of experiments. All experimental procedures and protocols have been approved by the Université Laval and University of Alberta Animal Care Committee in accordance with the guidelines detailed by the Canadian Council on Animal Care.

### Series I: Identification of Androgen Receptor mRNA in the Carotid Body and Nucleus of the Solitary Tract With RNAscope

The presence of androgen receptors in the CB was recently reported with immunohistochemistry (Marques et al., 2020). Here, we aimed to confirm the expression and distribution of androgen receptor mRNA within the CBs and caudal nucleus of the solitary tract (NTS) of three adult (P69) and three aging (P193-206) male rats using a complementary approach of *in situ* hybridization by RNAscope^®^ (Advanced Cell Diagnostics-ACD Bio, Newark, CA, United States). Rats were deeply anesthetized using urethane (1.5 mg/kg, i.p.) and perfused transcardially with 0.9% saline and 4% paraformaldehyde (PFA, 175 ml). Brains were extracted and post-fixed for 24 h in 4% PFA followed by 48 h in 30% sucrose (all 4°C). At the same time, left and right carotid bifurcations were removed and post-fixed in 4% PFA (4°C) for 2 h, washed 3 times (10 min) in phosphate buffered saline (1× PBS, in mM: 11.9 phosphate, 137 NaCl, 2.7 KCl; 21°C) and placed in 30% sucrose overnight (4°C). Tissues were frozen in Tissue-Tek O.C.T compound at −80°C and sectioned at 25–30 μm using a cryostat (CM1950, Leica, Buffalo Grove, IL, United States). Brain slices were washed in 1× PBS prior to mounting, while sections containing CBs were mounted directly onto slides (Superfrost Plus) and frozen at −80°C until processing. Slides were pre-treated on the morning of the assay as detailed in Biancardi et al. (2021) with the modification that CB slices were treated with H_2_O_2_ solution diluted by half. Following pre-treatment, slides were processed according to the manufacturer’s instructions (for CB’s Protease Plus was used in place of Protease III). Slides were then incubated with RNAscope oligonucleotide probe for nuclear androgen receptor (Rn-Ar-C1; #317141) for 2 h at 40°C. In parallel, medullary and bifurcation slices from each animal were treated with positive (low-copy housekeeping gene) and negative (non-specific bacterial gene) control probes provided by ACD Bio. Finally, slides were treated using the RNAscope Multiplex Fluorescent Assay Kit (V2). Probe RNA was visualized using Opal 570 reagent (1:1000; PerkinElmer, Woodbridge, ON, Canada). To visualize type I glomus cells comprising the CB, noradrenergic neurons of the NTS and choline acetyltransferase (ChAT) expressing neurons of the dorsal vagal complex, RNAscope slides were processed by immunohistochemistry for the staining of tyrosine hydroxylase (TH) and ChAT proteins by first blocking with 10% normal donkey serum + 0.3% triton-X 100 in 1× PBS for 60 min at 21°C. Slides were incubated with primary antibody overnight followed by secondary antibody for 2 h at room temperature: CB slides received anti-rabbit TH (1:1000; #AB152, EMD Millipore, Oakville, ON, Canada) followed by Cy5-conjugated donkey anti-rabbit IgG (1:200; Jackson Immuno Research Laboratories Inc., West Grove, PA, United States); medullary slides received anti-rabbit TH (1:1000; #AB152, EMD Millipore) plus anti-goat ChAT (1:500; #AB144P, EMD Millipore) followed by Cy2-conjugated donkey anti-rabbit IgG plus Cy5-conjugated donkey anti-goat IgG (1:200; Jackson Immuno Research Laboratories Inc.). Slides were rinsed with DAPI (ACDBio, diluted 1:2 with PBS, 45 s) and cover-slipped with Fluorsave mounting media (EMD Millipore). Representative high-resolution images (2048 × 2048) were taken with a Leica TCS SP8 Laser Scanning Confocal microscope (Concord, ON, Canada). Sections of the lateral septum from each male were labeled with the androgen receptor oligonucleotide probe to confirm probe efficacy (all showed strong fluorescence adjacent to the lateral ventricle, as expected). In all images, the DAPI signal was modified to decrease brightness and increase contrast using ImageJ software (Ver. 1.53e, NIH, United States). In the NTS, clusters of RNAscope signal (>8 “dots”) adjacent to a nucleus were interpreted as an androgen receptor-positive soma (arrows and arrowheads in Figure 2).

**FIGURE 1:**
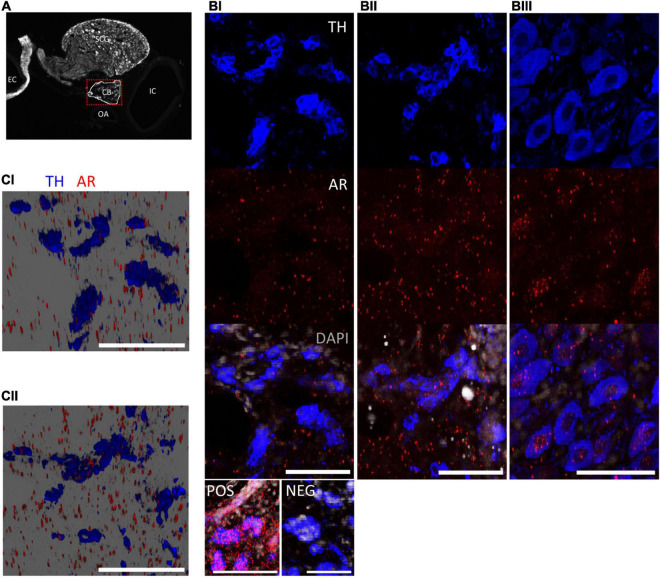
Androgen receptor mRNA is present at low levels in the carotid bodies. **(A)** Image of the carotid bifurcation (25 μm thick section) stained for tyrosine hydroxylase (TH); CB, carotid body; EC, external carotid artery; IC, internal carotid artery; OA, occipital artery; SCG, superior cervical ganglion. **(BI,II)** Representative confocal images of CBs show TH positive glomus cells (blue). Androgen receptor mRNA (AR, red) expression was weak in adult (i) and aging (ii) carotid tissue and rarely co-localized with glomus cells (images taken in ROI = red box). POS, NEG = positive and negative controls for RNAscope assay. **(BIII)** AR mRNA co-localized with TH-positive neurons of the SCG. **(CI,II)** 3D reconstruction of z-stacks from panels **(BI,II)** further suggest that while AR mRNA is expressed in the CB, it does not tend to occur in association with glomus cells of adult (i) or aging (ii) male rats. Scale bars = 50 μm.

**FIGURE 2:**
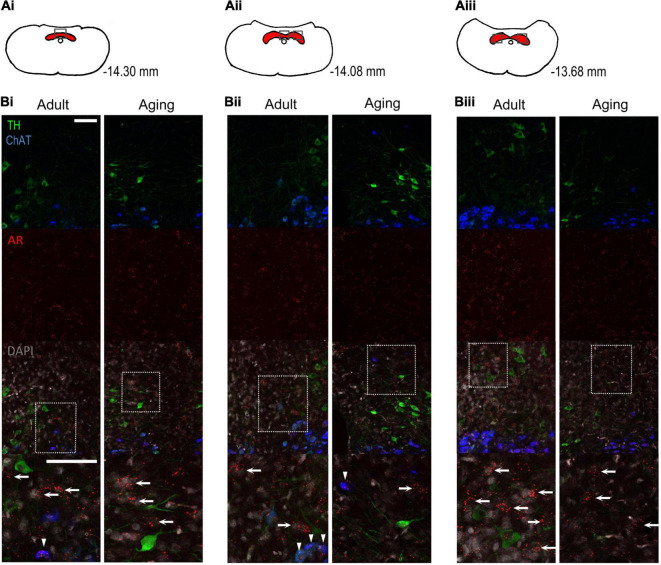
Androgen receptor mRNA is found in cells comprising the caudal NTS. **(AI–III)** Coronal sections from the rat caudal medulla showing the caudal NTS (red) and imaging locations (black boxes). Coordinates = mm from bregma. Adapted with permission from Paxinos and Watson (1998). **(BI–III)** Representative confocal images of the NTS at 25× in adult and aging male rats. ChAT-positive neurons comprise the dorsal vagal complex (arrowheads = co-localized with AR), while TH-positive neurons comprise the A2-region. Bottom panel = region of interest in merged image. AR mRNA is present in Chat/TH-negative cells (arrows) within the boundary of the NTS. *N* = 7 rats. Scale bars = 75 μm.

### Series II: Measurement of the Hypoxic Ventilatory Response in Anesthetized Rats

#### Animal Instrumentation

These experiments evaluated the effects of testosterone supplementation on the HVR under experimental conditions that facilitate standardization of hypoxic stimulation and quantification of arterial blood gases using standard procedures in our laboratory (Dumont and Kinkead, 2010). Anesthesia was induced by placing the rat in a closed chamber equilibrated with isoflurane (3.5%) and was then maintained *via* a nose cone (3%). The rat was placed on a homeothermic blanket (Harvard Apparatus, Holliston, MA, United States), and rectal temperature was maintained at 37°C. The trachea was cannulated, the nose cone was removed, and a “T”-shaped tube was placed on the endotracheal cannula and connected to the breathing circuit. Rats spontaneously breathed a mixture of 30% O_2_ and 70% N_2_ (FiO_2_ = 0.3) to maintain arterial blood gases within normoxic range (see Table 1). A venous femoral catheter was inserted to administer urethane and drugs. A second catheter was inserted in the femoral artery to monitor blood pressure (Transbridge TBM4M-B, World Precision Instruments, Sarasota, FL, United States) and to withdraw blood samples (70 μl) for analysis of arterial blood-gases (model ABL-5, Radiometer, Copenhagen, Denmark), corrected for the rat’s body temperature. Rats were slowly converted from isoflurane to urethane anesthesia (1.4 g/kg). The isoflurane level within the inspired gas mixture was decreased progressively, while urethane was infused slowly (0.125 ml/min) with a motorized pump (Harvard Instruments, Holliston, MA, United States; model PHD2000).

**TABLE 1:** Effects of acute testosterone propionate injection (2 mg/kg. i.p.) on basic cardiorespiratory variables measured in anesthetized rats (urethane; 1.4 g/kg) prior to hypoxic exposure.

	Baseline (normoxia)		Post-injection (30 min; normoxia)		Time effect	Injection effect	Age effect	Factorial interaction
	Vehicle (*n* = 6)	Testo (*n* = 7)	Vehicle (*n* = 6)	Testo (*n* = 7)				
Age (days)	86 ± 15	91 ± 14				NS		
Weight (g)	559 ± 56	505 ± 59				NS		
Breathing frequency (bursts min^–1^)	147 ± 19	137 ± 14	147 ± 18	134 ± 12	NS	NS	NS	NS
Burst amplitude (mV)	2.6 ± 0.9	2.3 ± 0.6	2.6 ± 0.8	2.3 ± 0.6	NS	NS	NS	NS
Body temperature (°C)	36.9 ± 0.4	36.0 ± 0.6^†^	37.1 ± 0.4	36.4 ± 0.3*	NS	*F*_(1_,_10)_ = 12.22 ***P* = 0.006**	NS	NS
Mean arterial pressure (mm Hg)	96 ± 22	112 ± 21	98 ± 21	116 ± 24	NS	NS	NS	NS
Heart rate (BPM)	311 ± 41	340 ± 38	322 ± 43	350 ± 35	NS	NS	NS	NS
PaO_2_ (mm Hg)	119 ± 13	133 ± 21	122 ± 13	127 ± 24	NS	NS	NS	NS
PaCO_2_ (mm Hg)	41 ± 3	44 ± 7	38 ± 6	41 ± 6	NS	NS	NS	NS
pHa	7.34 ± 0.03	7.31 ± 0.03	7.36 ± 0.03	7.35 ± 0.02	NS	NS	NS	NS

*Data are reported as means ± SD.*

* Different from corresponding baseline value at P < 0.05.

*^†^Different from corresponding vehicle value at P < 0.05.*

*Values in bold indicate a significant factor effect at P < 0.05.*

Electromyographic activity from the diaphragm (dEMG) was recorded as a correlate of inspiratory motor output. To do so, a ventral incision was performed to reach the diaphragm, and two stainless-steel electrodes were sewn into the diaphragm, 1 cm apart. The electrodes were placed as laterally as possible to reduce electrical interference from the heart. Electrical activity was amplified (gain = 10 000; model no. 1700, AM Systems, Everett, WA, United States), band-pass filtered (100 Hz to 10 kHz), and fed to a moving averager (time constant: 100 ms; CWE, model MA-821, Ardmore, PA, United States) before being digitized and recorded with a data acquisition system (IOX software, EMKA Technologies, Falls Church, VA, United States). Once the instrumentation was completed, the rat recovered for ∼30-min or until cardiorespiratory parameters were stable before experiments began.

#### Experimental Protocol

Baseline activity was recorded under normoxic conditions for 10 min. A first blood sample was withdrawn into a capillary tube to measure arterial blood gases at rest. A second sample was taken for analysis of hormone profiles. The rat then received either Depo-testosterone (testosterone cypionate; 100 mg/ml.; Pharmacia, New York, NY, United States. Dose: 2 mg/kg; *n* = 7) or a comparable volume of vehicle (∼1.5 ml; *n* = 6). Each ml of vehicle contained benzyl benzoate (0.1 ml) and alcohol benzoate (9 mg) dissolved in cottonseed oil. A 30 min period was allowed for drug absorption; this wait period is sufficient to observe clear effects on the rat’s behavior and cognition (Buddenberg et al., 2009). Blood samples were taken again to monitor blood gases and hormonal profile. Hypoxia was then induced rapidly by switching the inflowing gas to pre-mixed gas containing 12% O_2_ for 20 min. At the end of hypoxia, a final series of blood samples were taken. Euthanasia was performed by urethane overdose. All blood samples were placed in a serum-gel clotting activator microtube (Sarstedt, Nümbrecht, Germany) for analysis of progesterone, estradiol, and testosterone. Serum-gel tubes were kept at room temperature for 30 min before centrifugation (13,000 rpm, 4°C for 5 min). After centrifugation, blood serum was collected and placed in a −80°C freezer until assayed.

#### Data Analysis

dEMG bursts were detected by the acquisition system, and their amplitude was calculated as the difference between the peak and baseline activity; dEMG burst amplitude was used as an index of tidal volume. Owing to differences in contact efficiency and/or electrode placement, raw dEMG activity (in millivolts) was variable between experiments. To address this issue, the dEMG amplitude was expressed as a percentage change from baseline. Throughout the experiment, the dEMG amplitude, breathing frequency, mean arterial blood pressure, and heart rate data were averaged in 15-s bins. Prior to hypoxic stimulation, normoxic values were obtained by averaging 5 min of recording. During hypoxic stimulation, data were averaged every 2 min.

#### Hormone Assays

Analyses of total testosterone, estradiol, and progesterone were performed by Medical Biochemistry service of the Centre Hospitalier Universitaire de Québec (CHU de Québec, Québec, QC, Canada) using an electrochemiluminescence immunoassay test and read by the Elecsys 1010/2010 modular analyzer (Roche Canada, Mississauga, ON, Canada).

### Series III: Effects of Testosterone Supplementation on the Carotid Body’s Response to Hypoxia in *ex vivo* Preparations

#### *Ex vivo* Preparations

Rats were deeply anesthetized and both carotid bifurcations removed “en bloc.” Anesthesia was initiated with a single dose of ketamine/xylazine (0.1 ml/100 g, i.p.) and maintained using isoflurane (flow rate = 330–350 ml/min; room air plus 34–37% supplemental O_2_). Excised tissue was placed in a petri-dish containing ice-cold Tyrode solution (in mM: 125 NaCl, 5 KCl, 2 MgSO_4_, 1.2 NaH_2_PO_4_, 25 NaHCO_3_, 1.8 CaCl_2_, 5 sucrose, 10 glucose; pH 7.4) and oxygenated with carbogen (95% O_2_ + 5% CO_2_). The CSN was identified and the surrounding tissue gently cleaned away. Following dissection, the common carotid artery of *ex vivo* preparations was mounted onto an inflow catheter fitted inside a petri dish. Preparations were incubated at 32°C (Lab-Line Aquabath, Thermo Fisher, Ottawa, ON, Canada) with a concentration of “free” (i.e., bioavailable) testosterone above normal serum levels to mimic the high doses of testosterone replacement therapy administered clinically (5 nM of 4-Androsten-17β-ol-3-one; Sigma-Aldrich; Oakville, ON, Canada; *n* = 7 CBs from 5 adults + 7 CBs from 6 aging) or vehicle (DMSO < 0.001%; *n* = 7 CBs from 4 adults + 7 CBs from 6 aging) equilibrated with carbogen for 60–180 min. The details concerning the number of replicates and age range of each group are reported in Table 2. Excised bifurcations were expected to contain only minimal serum albumin or sex hormone binding globulins, both of which reversibly bind testosterone and contribute to “total” testosterone in intact animals. Our choice of concentration was therefore based on reported values of “free” testosterone in P55-75 male rats measured to be in the range of 0.3–1.5 nM (Nazian, 1986).

**TABLE 2:** Comparison of ages and body weights of animals used for *ex vivo* recording of carotid body activity.

	Adults		Aging	
	Control (*N* = 4; *n* = 7)	Testo (*N* = 5; *n* = 7)	Control (*N* = 6; *n* = 7)	Testo (*N* = 6; *n* = 7)
Age (days)	70 ± 4	73 ± 4	185 ± 4	187 ± 5
Weight (g)	476 ± 48	544 ± 27	1003 ± 66	948 ± 93

*Data are reported as mean ± SEM.*

#### Extracellular Recording of Carotid Sinus Nerve Activity

Carotid sinus nerve activity was recorded using standard protocols (Soliz et al., 2016). Briefly, the common carotid artery was continuously perfused with Tyrode solution equilibrated with carbogen at a rate of 10 ml/min. All solutions were maintained at 36–38°C using an Isotemp 3013HD circulator (Fisher Scientific, Ottawa, ON, Canada) and continuously monitored (Thermalert TH-5, GENEQ Inc., Montreal, QC, Canada). Extracellular recordings were obtained by drawing the CSN into the tip of a borosilicate glass electrode (0.84 mm i.d.) pulled to a fine tip with a vertical puller (Stoelting Instruments, Wood Dale, IL, United States). A grounding electrode was placed in the recording chamber and a reference tip inserted into the tissue immediately proximal to the bifurcation.

#### Experimental Protocol and Data Analysis

Experiments commenced when a stable neural signal was achieved (∼20 min). The neural signal was fed to a differential input head-stage pre-amplifier (Neurolog NL100AK), filtered (100–1500 Hz), and amplified (Neurolog modules, NL104A, NL126, NL106; Warner Instruments, Hamden, CT, United States). A Micro 1401 mk II A/D converter (Cambridge Electronic Design Ltd. (CED), Cambridge, United Kingdom) was used to digitize the signal for display of raw and integrated activity using Spike 2 software (CED). Baseline activity was normalized to 5 impulses/sec using the Spike 2 software prior to recording. Chemoreceptor-related discharges were discriminated using Spike 2 as action potentials with amplitude of 25% above baseline noise, and which were responsive to a decrease in perfusate PO_2_ with a reversible increase in discharge frequency.

Comparison of carotid body function in adult and aging rats was assessed as follows: the CNS discharge rate was recorded under baseline conditions for 5 min during continuous perfusion with Tyrode solution equilibrated with 5% CO_2_ + 95% O_2_. The perfusate was then switched to Tyrode solution equilibrated with a hypoxic gas mixture (5% CO_2_ + 95% N_2_) for 15 min, after which the preparation was returned to hyperoxic/normocapnic conditions for 10 min. The protocol terminated with perfusion of 5 mL of a high K^+^ solution to maximally activate chemoreceptors. All solutions were delivered at a rate of 10 ml/min.

### Series IV: Impacts of Testosterone Supplementation on Apneas in Freely Behaving Rats

This series of experiments aimed to: (1) evaluate the impacts of testosterone supplementation on the functionality of respiratory control in naturally sleeping animals and (2) determine whether the effects are age-dependent. The occurrence of apneas was recorded using a non-invasive approach. Experiments were performed on three age groups: 80 days old (82 ± 1.8 days), 120 days old (121 ± 2 days), and 200 days old (182 ± 6.5 days). These age groups do not perfectly align with those used in the previous series of experiments but because they reflect distinct age-stages in sexual function and testosterone levels, they allow a more complete assessment of the functional impact of testosterone supplementation in intact rats.

#### Experimental Protocol

Experiments began by injecting rats with vehicle according to the protocol described in series II (anesthetized rats), the rat was then placed in the 5L Plexiglas plethysmography chamber where it was allowed to acclimatize under normoxic conditions for approximately 30 min. Once the rat appeared calm and its respiratory signal was stable with minimal movement artifacts, ventilatory activity was then recorded for 1.5–2 h using a whole-body, flow- through plethysmograph (PLY3223; Buxco Electronics, Sharon, CT, United States) as reported previously (Fournier et al., 2015). After this initial trial, animals received an IP injection of Depo-testosterone (2 mg/kg; Pharmacia, New York, NY, United States). To be consistent with the approach used in Series II, a 30 min wait period was allowed for drug absorption and effect before plethysmography recording was re-initiated for an additional 1.5–2 h. Recordings were performed between 10 AM and 5 PM, when rats normally sleep. We opted for this protocol because it makes it possible to compare the effects of vehicle and testosterone injection on the same animal and thus reduce the number of rats tested. However, because the actions of testosterone are prolonged in time, it was not possible to determine the order randomly. While respiratory variables and metabolism of the rat remain stable over the period during which the experiments were performed (Mortola and Seifert, 2002), the potential impact on of the ∼3 h delay between vehicle vs. testosterone measurements on sleep architecture and apnea are unknown. This limitation will be considered in data interpretation.

#### Data Analysis

Quantification of apneas was performed on 1 h of respiratory traces when ventilation was stable and the rat was in non-REM sleep; comparing variability of respiratory activity with changes EEG/EMG recordings across sleep wake states, Bastianini et al. (2017) showed that non-REM sleep can be reliably identified owing to the high stability of breathing frequency and tidal volume. Specifically, comparing of the identification of sleep/wake states with this non-invasive method with the classical approach involving EEG and EMG monitoring shows that the two methods agree 90% of the time (Bastianini et al., 2017). A non-invasive approach obviates concerns related to confounding effects of the stress associated with surgical implantation of EEG–EMG electrodes. Apneas were identified according to standard criteria, which define an apnea as the absence of respiratory flow for at least two normal breathing cycles (Montandon et al., 2006). Apneas were then categorized depending on whether they occurred following a sigh (post-sigh) or spontaneously (interruption of normal breathing activity, without preceding sigh). Sighs were defined as an inspiratory effort with an amplitude at least 2 times greater than the normal tidal volume (Marcouiller et al., 2014). Data were then compared within subjects (vehicle vs. testosterone injection) and between age groups.

### Statistics

**FIGURE 3:**
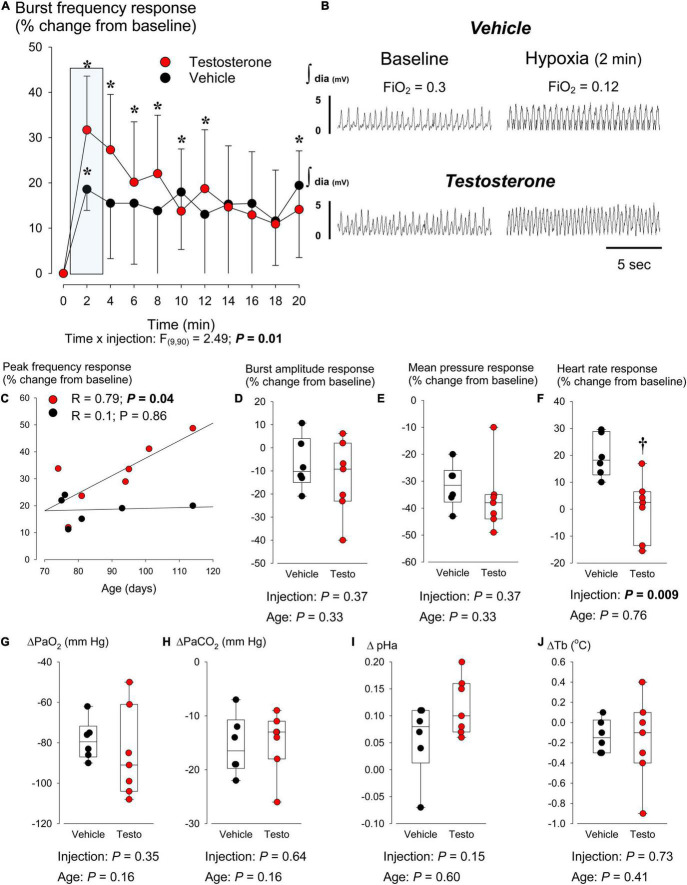
Testosterone supplementation potentiates the acute hypoxic ventilatory response. **(A)** Comparison of the time course of the breathing frequency response to hypoxia between rats supplemented with testosterone propionate (red circles; 2 mg/kg; i.p.) or vehicle (black circles; benzyl benzoate + alcohol benzoate dissolved in cottonseed oil) 30 min prior to the onset moderate hypoxia (FiO_2_ = 0.12; 20 min). The response is expressed as a percent change from baseline (FiO_2_ = 0.30, balance N_2_). Rats were anesthetized with urethane (1.4 g/kg; i.v.). Respiratory activity was measured by placing and electromyogram (EMG) electrode in the diaphragm. **(B)** Representative EMG recordings comparing inspiratory activity under baseline condition and 2 min after the onset of hypoxia between rats that received vehicle (top traces) and testosterone (bottom traces). **(C)** Correlation analysis of the relationship between the rat’s age (days) and the peak increase in breathing frequency in response to hypoxia (2 min). Correlations are compared between rats treated with vehicle (black circles) or testosterone (red circles). Box plots comparing **(D)** EMG amplitude, **(E)** mean arterial blood pressure, and **(F)** heart rate responses measured at the end of the hypoxic stimulus; these results are expressed as a percent change from baseline. Changes in **(G)** arterial PO_2_, **(H)** arterial PCO_2_, **(I)** arterial pH, and **(J)** body temperature relative to baseline; these results are expressed in absolute values. In each box plot, the boundary of the box closest to zero indicates the 25th percentile, the line within the box marks the median, and the boundary of the box farthest from zero indicates the 75th percentile. Whiskers (error bars) above and below the box indicate the 90th and 10th percentiles. * indicates value different from baseline at *P* < 0.05. † indicates a value different from vehicle at *P* < 0.05.

**FIGURE 4:**
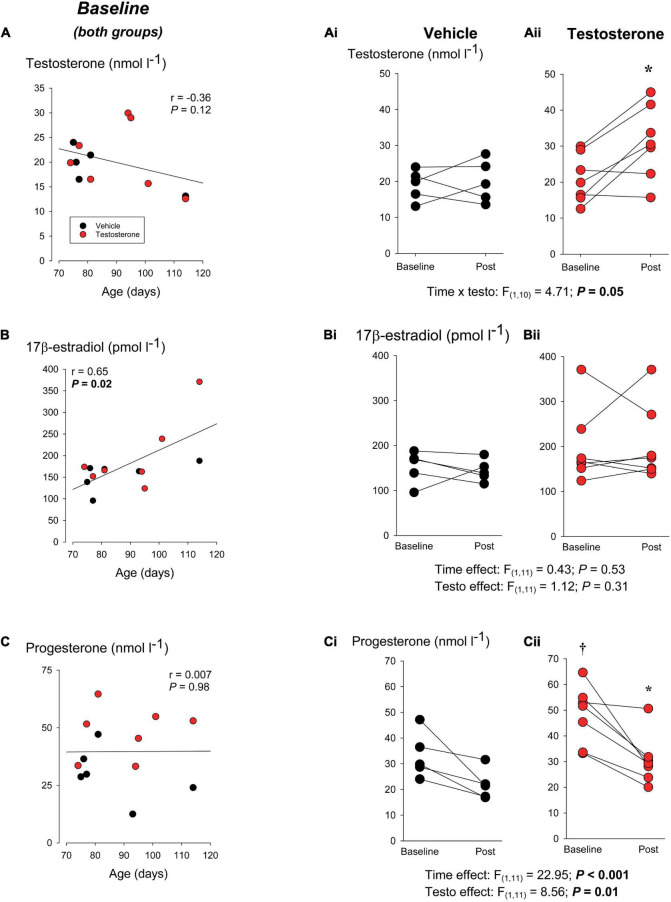
Hormone profile of rats before and after testosterone supplementation. Left hand panels show basal levels of **(A)** testosterone, **(B)** 17β-estradiol, and **(C)** progesterone across the age-range of the rats used in the first series of experiments (anesthetized rats). Blood samples were obtained from rats assigned to receive an i.p. injection of vehicle (black circles) or testosterone propionate (red circles; 2 mg/kg; i.p.). Samples were taken under basal conditions (FiO_2_ = 0.30, balance N_2_) before animals received their injection. The panels on the right show the effect of injecting **(Ai,Bi,Ci)** vehicle or **(Aii,Bii,Cii)** testosterone for each hormone. Baseline levels are the same as those reported on the left but for each animal, the “post” values were measured at the end of the protocol; i.e., after hypoxic exposure (FiO_2_ = 0.12; 20 min). * indicates a value significantly different from corresponding baseline value at *P* < 0.05; † indicates a value significantly different from the corresponding vehicle value at *P* < 0.05.

**FIGURE 5:**
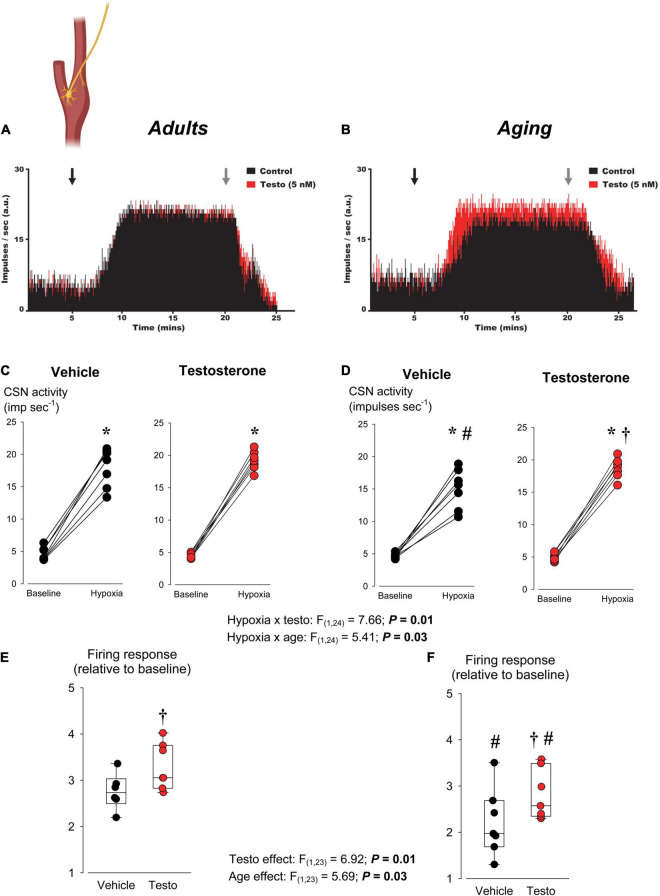
Effects of age and testosterone on the carotid body’s response to hypoxia. **(A)** Representative electroneurograms (ENG) illustrating the rise in carotid sinus nerve (CSN) activity when the medium used to perfuse an *ex vivo* carotid body preparation is changed from normoxia (95% O_2_ + 5% CO_2_) to hypoxia (0% CO_2_ + 5% CO_2_, balance N_2_). Arrows indicate the time when medium is changed. Results were obtained from adult rats (∼P70) preparations incubated either with vehicle (<0.001% DMSO; black ENG) or testosterone (5 nM; red ENG) 90–120 min prior to the onset of the experiments. Note that in those experiments, the responses (vehicle vs. testosterone) were nearly identical and the ENGs overlap. **(B)** Neurograms as in panel **(A)** comparing the effect of testosterone incubation on the responses from preparations obtained from aging rats (∼180 days old). Individual CSN activity (impulses sec^– 1^) from preparations from **(C)** adult or **(D)** aging rats under baseline conditions and during hypoxia; preparations were incubated with vehicle (black circles) or testosterone (red circles). Box plots show normalized firing rate responses (expressed as “fold-change” from baseline) for preparations from **(E)** adult and **(F)** aging rats incubated with vehicle (black circles) or testosterone (red circles). In each box plot, the boundary of the box closest to zero indicates the 25th percentile, the line within the box marks the median, and the boundary of the box farthest from zero indicates the 75th percentile. Whiskers (error bars) above and below the box indicate the 90th and 10th percentiles. *N* = 7 rats per group. * indicates a mean value significantly different from corresponding baseline value at *P* < 0.05; † indicates a mean value significantly different from the corresponding vehicle value at *P* < 0.05. # indicates a mean value significantly different from the corresponding adult value at *P* < 0.05.

**FIGURE 6:**
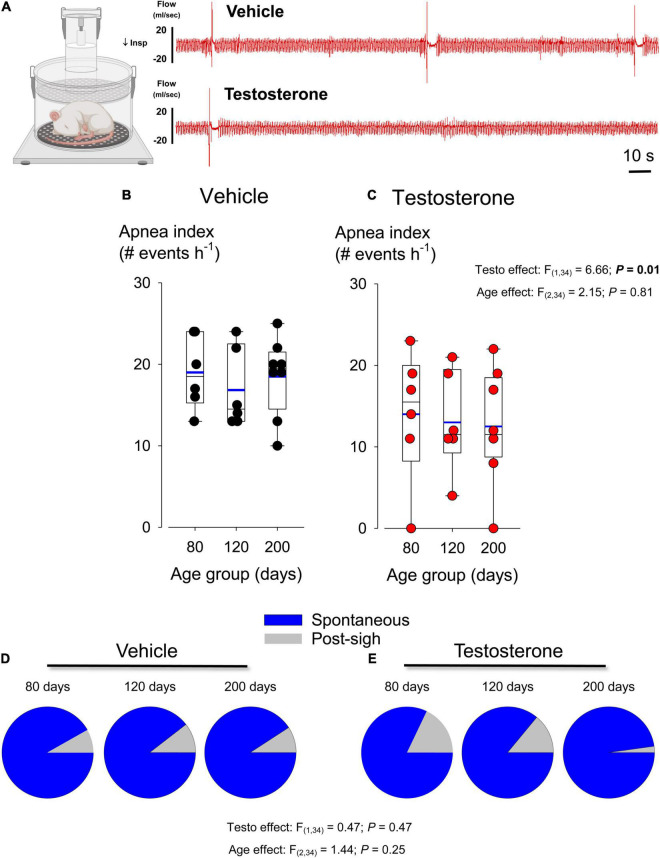
Testosterone supplementation reduces apneas during sleep. **(A)** Comparison of plethysmography recordings obtained from adult rats (80 days old) that either received an intraperitoneal injection of vehicle (top trace; benzyl benzoate + alcohol benzoate dissolved in cottonseed oil) or testosterone propionate (lower trace; 2 mg/kg; i.p.) 30 min prior to the onset of the experiment. Sleep/wake states was assessed according to the stability of the respiratory signal as described by Bastianini et al. (2017). **(B)** Box plots comparing the apnea index of vehicle-treated rats at three distinct age groups: 80, 120, and 200 days old. **(C)** Similar comparison in testosterone-treated rats. The apnea index is defined as the number of apneic events per hour of sleep. In each box plot, the boundary of the box closest to zero indicates the 25th percentile, the line within the box marks the median, and the boundary of the box farthest from zero indicates the 75th percentile. Whiskers (error bars) above and below the box indicate the 90th and 10th percentiles. **(D)** Pie charts comparing the proportion of spontaneous (blue) and post-sigh (gray) apneas measured in vehicle-treated rats at 80, 120, and 200 days of age. **(E)** Similar comparison in testosterone-treated rats. See section “Materials and Methods” for the details on the criteria used to identify apnea type.

Results were analyzed using a multifactorial ANOVA for the effect of age, testosterone treatment (Figures 5, 6); time-dependent changes was tested also (Figure 3). A repeated measures design was used when appropriate. When ANOVA results indicated that a factor was significant (*P* < 0.05), a Fisher’s least significant difference test was performed for *post hoc* analysis. The relationships between age and the hypoxic response (Figure 3) or the hormone levels (Figure 4) were assessed by Pearson’s correlation analyses. Statistical analyses were performed using JASP (ver 0.14.1 University of Amsterdam, Netherlands). Normality and equality of variance was tested in all analyses using the Shapiro-Wilk and Levene’s tests, respectively. All data passed those tests. Data are reported as mean ± standard deviation (SD). ANOVA results are mainly reported in the text and Table 1. Results from *post hoc* tests are represented by symbols in the table and figures.

## Results

### Series I: Androgen Receptor mRNA Is Expressed at Low Levels in the Carotid Body and Nucleus of the Solitary Tract

Considering that previous studies have shown that testosterone alters CB function and that previous reports using immunohistochemistry suggest ARs are present within the carotid body (Marques et al., 2020), we hypothesized that chemoreceptive glomus cells of the CB’s would express nuclear AR mRNA. Accordingly, we used *in situ* hybridization to demonstrate AR mRNA in adult (P69) and aging (P193-206) male rats. The carotid body is a heterogeneous organ comprised of glomus cells, glial-like cells, petrosal afferents and extensive vascularization (Leonard et al., 2018). Histological data presented in Figure 1 indicates that AR mRNA was present within the carotid body tissue of adult and aging male rats, however, expression levels were low when compared to the positive control (Figure 1BI bottom panel) and expression did not co-localize with TH-positive glomus cells (Figures 1A,BI,II,CI,II). It is possible that AR mRNA instead co-localizes with glial-like cells, petrosal afferents or vasculature in the organ, although we did not test this. The AR mRNA did however co-localize with TH-positive neurons in the superior cervical ganglion, part of the sympathetic nervous system (Figure 1BIII).

Based on the modest levels of AR mRNA within the CB’s, we next determined whether neurons of the caudal NTS showed AR mRNA. This medullary region was analyzed because it is the primary projection site of CB sensory afferents (Finley and Katz, 1992). Consistent with previous results using immunohistochemistry (Fournier et al., 2014), cells comprising the commissural and medial NTS showed AR mRNA expression in adult and aging rats (Figures 2AI–III). These cells were ChAT- and TH-negative, indicating they did not comprise the dorsal vagal complex or A2 region (Figures 2AI–III, arrows). We noted that ChAT-positive neurons of the dorsal vagal complex also expressed modest levels of AR mRNA in the cytoplasm and nucleus (Figures 2AI–III, arrowheads). Regardless of age, all rats showed strong cytoplasmic and nuclear AR mRNA expression in neurons of the lateral septum, which are known to express ARs (data not shown). While we did not determine AR protein expression *per se*, the localization of AR mRNA in the cytoplasm suggests translational potential for protein synthesis. Taken together, these results support an anatomical basis for testosterone modulation of the carotid body sensory pathway in adult and aging male rats.

### Series II: Age-Dependent Potentiation of the Hypoxic Ventilatory Response by Testosterone Supplementation in Anesthetized Rats

Following identification of AR mRNA in the CB’s and NTS, we questioned whether *in vivo* testosterone supplementation would augment the HVR in anesthetized animals wherein changes in arterial blood gasses could be monitored and cardiovascular parameters could be measured. Under baseline conditions, age, body weight, and cardiorespiratory variables measured in vehicle and testosterone-injected rats were similar (Table 1). Testosterone injection resulted in a 0.4°C rise in Tb; none of the other physiological variables were altered by treatment. Figure 3A compares the time course of the HVR in vehicle vs. testosterone-injected rats. Exposure to hypoxia initiated a rapid rise in breathing frequency that peaked at 2 min and declined slightly thereafter. The response measured in testosterone-injected rats was 13% greater than in animals that received vehicle (Figures 3A,B). Considering the animal’s age in the analysis showed that the intensity of the peak frequency response measured in testosterone-treated rats was positively correlated with increasing age of the animal. This relationship was not observed in vehicle-treated rats (Figure 3C). The burst amplitude and mean blood pressure responses to hypoxia were not affected by testosterone injection (Figures 3D,E). However, testosterone injection reduced the heart rate response by 19% (Figure 3F). The changes in arterial blood gases and Tb induced by hypoxia did not differ between groups (Figures 3G–J).

We then compared the basal (pre-injection) age-related changes in the hormonal profile of rats assigned to each group. Total testosterone tended to decrease, whereas 17β-estradiol augmented significantly with age; progesterone remained unchanged. The panels shown at the right in Figure 4 compare pre- vs. post-injection values for each hormone. Baseline testosterone did not differ between groups and injection augmented circulating levels only in testosterone-treated animals (Figures 4AI,II); the levels achieved post-supplementation were slightly higher than those reported in males following hypoxic exposure (Fournier et al., 2014; Tenorio-Lopes et al., 2017) or the peak observed at sexual maturity (P65) (Nazian, 1986). 17β-estradiol was similar between groups and was not altered by testosterone injection (Figures 4BI,II). Prior to injection, progesterone levels of rats assigned to receive testosterone were 61% greater than the vehicle group. Levels measured at the end of the experiment were lower than at rest, but the magnitude of the decrease did not differ between groups (Figures 4CI,II).

### Series III: Testosterone Augments Carotid Body Responsiveness to Hypoxia

Our data from Series II demonstrated that augmentation of the HVR by testosterone was most important for the peak frequency response occurring within 2 min of exposure. Since the CB’s contribute to the peak hypoxic response (Powell et al., 1998), we postulated that testosterone would act on these organs to potentiate CSN activity. Exposing *ex vivo* CB’s to hypoxic superfusate augmented CSN activity in all preparations (Figures 5A–D). Whether expressed as absolute values (Figures 5C,D) or normalized to baseline (Figures 5E,F), the hypoxic response recorded from preparations made from aging rats was 0.5 fold lower than that measured in adults. When normalized to baseline, testosterone augmented the firing rate observed during hypoxia equally in adult vs. aged rats, indicating that the treatment effect was not age-dependent (Figures 5E,F). Interestingly, since the hypoxic responsiveness of aging CBs was blunted relative to adults, incubation of the aging CB with testosterone increased the magnitude of the hypoxic response to that comparable to vehicle treated adult males (compare Figure 5D, right to Figure 5C, left).

### Series IV: Testosterone Reduces the Incidence of Apneas in Intact, Naturally Sleeping Rats

Augmented carotid body responsiveness to hypoxia has been proposed to contribute to respiratory instability and apnea during sleep. However, comparison of plethysmography recordings between groups illustrated that testosterone treatment reduced the apneic index during non-REM sleep (Figure 6A). Group data support this observation and show that, on average, the apnea index of testosterone treated rats was 27% less than vehicle; this effect was not influenced by the age of the animal (Figures 6B vs. 6C). The majority of the apneas were post-sigh and neither treatment nor age altered the post-sigh/spontaneous ratio significantly (Figures 6D,E). The duration of apneas was unaffected by age or treatment (*F*_(2,34)_ = 0.640; *P* = 0.53 and *F*_(1,34)_ = 0.98; *P* = 0.33, respectively; data not shown).

## Discussion

In men, the age-related drop in testosterone contributes to a progressive decline in physical and cognitive performances (Decaroli and Rochira, 2017). Testosterone supplementation generally alleviates the consequences of aging (Williams and Cho, 2017), but in patients suffering from SDB, this treatment has been reported to increase the apnea/hypopnea index with degradation of the O_2_ desaturation/hypoxemia profile (Sandblom et al., 1983; Hoyos et al., 2012). Liu et al. (2003) report worsening of the apneic index and O_2_ desaturation during NREM sleep in testosterone-treated aged men, but this was not due to decreased upper airway caliber; alluding instead to altered neural control mechanisms (Liu et al., 2003). An excessive hypoxic chemoreflex is an important mechanism in the pathophysiology of SDB (Smith et al., 2003; Younes, 2008; Ortega-Sáenz and López-Barneo, 2020), and there is growing evidence indicating that testosterone can augment the HVR; ARs are expressed in the CB’s and its main central target: the NTS (Fournier et al., 2014; Marques et al., 2020). In addition, testosterone supplementation in cats was shown to potentiate the carotid body’s responsiveness to hypoxia, but the use of neutered animals and the high testosterone levels achieved in those experiments do not allow a firm conclusion on the hormone’s action on O_2_ chemoreceptors (Tatsumi et al., 1994). To address this important issue, we tested the hypothesis that testosterone incubation potentiates the carotid body’s response to hypoxia and determined whether these effects were age-dependent. The age range of the animals used in Series II was not sufficiently large to show a significant drop in total testosterone. Although data variability/low number of replicates contributes to this outcome, we nonetheless broadened the age range of the rats used in the other series of experiments to increase the likelihood that age-related drop in testosterone was statistically (and physiologically) significant. Furthermore, we ensured that the levels achieved with the acute incubation protocol resulted in bioavailable testosterone above normal range (i.e., incubation media = 5 nM vs. normal rage of free testosterone = 0.43 ± 0.14 nM in adult rats) and in line with high-dose testosterone replacement therapy. We then furthered our basic understanding of the actions of testosterone on respiratory control by testing the effects of testosterone supplementation on the HVR and occurrence of apneas in intact rats. We show that testosterone supplementation potentiates the HVR, especially in older rats. While testosterone augmented the carotid body’s O_2_ response, this effect was marginal and occurred with equal magnitude in both age groups tested. Identification of AR mRNA in brainstem regions known to receive carotid body afferents suggests that central androgen signaling may be more important for augmentation of the HVR, especially in aging males. As a whole, our data suggest the potential for beneficial outcomes of testosterone supplementation as our protocol stabilized breathing during sleep.

### The Time Domains of Androgen Signaling

Androgens are mainly known to exert genomic effects *via* nuclear receptors. This classical mode of action is relatively slow, since translocation of bound androgen receptors from the cytoplasm to the nucleus takes ∼20 min (Chiu et al., 2016) and subsequent genomic effects have been reported within 1 h (Jones et al., 2003). This contrasts with the “rapid response” in which testosterone activates membrane bound receptors that trigger second messenger signaling ultimately regulating membrane-bound ion channels (Rosner, 1999). Testosterone-mediated vasodilatation is evident within minutes of application and is maximal by 20 min, thus indicating that activation of protein synthesis is not a pre-requisite for this action (Jones et al., 2003). These distinct modes of action (membrane vs. nuclear) allow testosterone to act on a broad timescale; however, clinical evaluations of the impacts of testosterone supplementation are generally performed on patients that have been treated for several weeks, thus allowing sufficient time for the hormone to induce both genomic and acute responses throughout the organism. Animal studies most relevant to the present study have been performed in neutered cats and although the details of the supplementation protocol are not reported, the use of subcutaneous pellets is indicative that the protocol lasted at least 1 week (Tatsumi et al., 1994). Subjecting rats to a chronic protocol may have ensured full expression of the genomic effects; however, the approach used here allowed us to observe the onset of a genomic response while the acute effect of testosterone was still fully preserved. While it may not be possible to ascribe the changes observed to one specific mode of action, the data reported here nonetheless provide highly valuable insights into testosterone’s actions on respiratory control.

### Age-Dependent Potentiation of the Hypoxic Ventilatory Response by Testosterone Supplementation

In an intact animal, the rapid rise in breathing frequency that takes place at the onset of hypoxia is initiated by the CB’s (Powell et al., 1998). Results showing that, in anesthetized rats, the peak in frequency response was greater following testosterone treatment provided preliminary support to our hypothesis, thus justifying further experiments. However, the data variability observed in testosterone treated rats was ∼3 times larger than in rats that received vehicle. While the age range of the rats was initially deemed small, subsequent analyses showed that it was sufficient to reveal age-dependent effects in the acute phase of the HVR and that age-related decline in testosterone level (and its conversion into 17β-estradiol) had begun. As a result, the effect of age was assessed in all analyses for this series and distinct groups of animals covering a larger age range was used for subsequent experiments that initially focused on the CB’s.

Using a stimulation protocol similar to the one used here, Conde et al. (2006) reported that carotid body responsiveness to hypoxia declines with age. Using 3 month-old rats as a reference, they first measured the response at 1 year and showed that the rise in CSN activity was ∼2 fold lower; this decline in responsiveness continues until 18 months and stabilizes thereafter. Here, the 0.5 fold drop in response obtained in our “aged” (∼6 months old) rats is consistent with their report. Our data showing that incubating CB’s with testosterone “restores” the O_2_ response of older rats to the level observed in young adults points to an important role of testosterone in that age-dependent drop in sensitivity. However, this stimulatory action of testosterone did not increase with age. This observation contrasts with age-dependent potentiation of the HVR observed in intact rats, thus indicating that testosterone’s actions within the central nervous system likely contributes to this process.

### Testosterone Supplementation Stabilizes Breathing During Sleep

Patients suffering from SDB breathe normally during the day and the loss of wakefulness drive to breathe reveals dysfunction in neural control. Accordingly, our results showing that testosterone potentiates the HVR in older rats and the CB’s response to hypoxia brought us to predict that testosterone supplementation would augment respiratory instability in naturally sleeping rats. While the effects of our protocol on the apnea index were modest, they nonetheless contradict our hypothesis and show that our testosterone supplementation protocol stabilizes breathing during sleep. As testosterone was injected systemically, the mechanism responsible for this effect is unknown; however, a combination of central and peripheral effects is likely. The experiments performed here do not allow us to explain the origins of this discrepancy; however, the current literature provides plausible explanations.

Chronic stress, including that resulting from SDB, inhibits the gonadotropic axis and clinical data show that testosterone levels in SDB patients are lower than age-matched controls (Luboshitzky et al., 2005). Furthermore, the testosterone/cortisol (T/C) ratio measured in patients with severe obstructive sleep apnea (OSA) is lower than those with moderate vs. severe OSA (Mohammadi et al., 2019). A similar drop in basal corticosterone level has been reported in rats previously subjected to neonatal maternal separation (NMS), a form of early life stress causing chronic activation of the corticotropic axis in animals and humans (Tenorio-Lopes and Kinkead, 2021). Of note, adult males (but not females) subjected to NMS show greater respiratory instability during sleep than controls (Kinkead et al., 2009); an augmented HVR and carotid body function contribute to this abnormal phenotype (Genest et al., 2004; Soliz et al., 2016). However, gonadectomy reduces the HVR of NMS rats but not controls (Fournier et al., 2014). Together, these data indicate that stress alters testosterone production and its actions on respiratory control. Given that our experiments were performed on animals receiving standard care (i.e., unstressed), testing the effects of testosterone supplementation on stressed rats would be informative in that regard.

## Conclusion and Perspectives

Sex hormones are often ascribed to sexual behaviors, however, their broad roles in physiological processes are now beginning to be realized. The relationship between testosterone and respiratory control is complex and not yet completely understood. In particular, the peripheral and central nervous systems express ARs in key areas involved in respiratory control; yet the contribution of these receptors to respiratory behaviors remains unresolved. Our data indicating that testosterone supplementation potentiates the carotid body’s response to hypoxia support our initial hypothesis but the results obtained in sleeping rats does not indicate that that it augments the occurrence of apneas, at least in healthy animals. Given the strong sex bias of respiratory diseases such as sleep disordered breathing further research into the consequences of sex hormone-mediated cellular signaling is warranted.

## Data Availability Statement

The raw data supporting the conclusions of this article will be made available by the authors, without undue reservation.

## Ethics Statement

The animal study was reviewed and approved by Animal Care Committee of Université Laval, Health Science Animal Policy and Welfare Committee of University of Alberta.

## Author Contributions

TJ, RK, VJ, and JS conceived and designed the experiments. TJ, DA-M, and SF performed the experiments. TJ, DA-M, SF, and RK analyzed and interpreted the data. TJ and RK wrote the manuscript. All authors approved the final version of the manuscript.

## Conflict of Interest

The authors declare that the research was conducted in the absence of any commercial or financial relationships that could be construed as a potential conflict of interest.

## Publisher’s Note

All claims expressed in this article are solely those of the authors and do not necessarily represent those of their affiliated organizations, or those of the publisher, the editors and the reviewers. Any product that may be evaluated in this article, or claim that may be made by its manufacturer, is not guaranteed or endorsed by the publisher.
