# Corrigendum: Testosterone Supplementation Induces Age-Dependent Augmentation of the Hypoxic Ventilatory Response in Male Rats With Contributions From the Carotid Bodies

**DOI:** 10.3389/fphys.2022.964673

**Published:** 2022-07-08

**Authors:** Tara A. Janes, Danuzia Ambrozio-Marques, Sébastien Fournier, Vincent Joseph, Jorge Soliz, Richard Kinkead

**Affiliations:** ^1^ Department of Physiology, Women and Children’s Health Research Institute, University of Alberta, Edmonton, AB, Canada; ^2^ Department of Pediatrics, Québec Heart and Lung Institute, Université Laval, Québec, QC, Canada; ^3^ Department of Surgery, Faculty of Medicine, Université Laval, Québec, QC, Canada

**Keywords:** carotid body, hypoxia, testoserone, sleep apnea, respiration, nucleus of the solitary tract

In the published article, there was an error in the **Funding** statement. The Canadian Lung Association funding information was mistakenly omitted. The corrected **Funding** statement appears below.

“TJ received a studentship from the scholarship supplement program of the Québec respiratory Health Research Network and Canadian Lung Association BAO Fellowship. DA-M was the recipient of the scholarship FRQS 268346 from the “Fonds de Recherche du Québec–Santé.” Research conducted at University of Alberta was supported by the Canadian Lung Association, Grant In Aid program awarded to S. Pagliardini. Research conducted at Université Laval was supported by the Canadian Institutes of Health Research (CIHR, grant #PJT-437541) awarded to RK.”

The authors apologize for this error and state that this does not change the scientific conclusions of the article in any way. The original article has been updated.

